# Correction: Sodium taurocholate cotransporting polypeptide is a functional receptor for human hepatitis B and D virus

**DOI:** 10.7554/eLife.05570

**Published:** 2014-11-20

**Authors:** Huan Yan, Guocai Zhong, Guangwei Xu, Wenhui He, Zhiyi Jing, Zhenchao Gao, Yi Huang, Yonghe Qi, Bo Peng, Haimin Wang, Liran Fu, Mei Song, Pan Chen, Wenqing Gao, Bijie Ren, Yinyan Sun, Tao Cai, Xiaofeng Feng, Jianhua Sui, Wenhui Li

Yan H, Zhong G, Xu G, He W, Jing Z, Gao Z, Huang Y, Qi Y, Peng B, Wang H, Fu L, Song M, Chen P, Gao W, Ren B, Sun Y, Cai T, Feng X, Sui J, Li W. 2012. Sodium taurocholate cotransporting polypeptide is a functional receptor for human hepatitis B and D virus. *eLife* **1**:e00049. doi: 10.7554/eLife.00049. Published 13 November 2012

There was an error in the section ‘Quantification of HBV cccDNA by qPCR’ in the ‘Materials and methods’. The nucleotide sequence of primer ccc-2316R was from the sense strand and wrongly given as 5′-GACCACCAAATGCCCCT-3′. Its reverse complementary sequence, 5′-AGGGGCATTTGGTGGTC-3′, is the correct 5′->3′ sequence of this primer. We thank Timothy Sheahan (GSK) for bringing this to our attention.

The article has been corrected accordingly.

