# Correction: Sodium taurocholate cotransporting polypeptide is a functional receptor for human hepatitis B and D virus

**DOI:** 10.7554/eLife.106838

**Published:** 2025-03-21

**Authors:** Huan Yan, Guocai Zhong, Guangwei Xu, Wenhui He, Zhiyi Jing, Zhenchao Gao, Yi Huang, Yonghe Qi, Bo Peng, Haimin Wang, Liran Fu, Mei Song, Pan Chen, Wenqing Gao, Bijie Ren, Yinyan Sun, Tao Cai, Xiaofeng Feng, Jianhua Sui, Wenhui Li

**Keywords:** Viruses, Other

Yan H, Zhong G, Xu G, He W, Jing Z, Gao Z, Huang Y, Qi Y, Peng B, Wang H, Fu L, Song M, Chen P, Gao W, Ren B, Sun Y, Cai T, Feng X, Sui J, Li W. 2012. Sodium taurocholate cotransporting polypeptide is a functional receptor for human hepatitis B and D virus. *eLife* **1**:e00049. doi: 10.7554/eLife.00049.

Published 13 November 2012

It has come to our attention that there is an image error in Figure 7A, where representative Delta Ag staining images of HDV-infected Huh-7 cells expressing wild-type NTCP receptors from three species —human (h), treeshrew (ts), and monkey (mk) —as well as several hNTCP and mkNTCP chimeric mutants, are shown.

The error is that the image for Delta Ag*hNTCP mk157-165 (the 2^nd^ image from the left in the 4^th^ row) partially overlaps with that of Delta Ag*mkNTCP Q84R/N86K (the 4^th^ image from the left in the 4^th^ row) in Figure 7A. The latter image was found to be mistakenly cropped from the source image of the former. Both the “hNTCP mk157-165” and “mkNTCP Q84R/N86K” receptor mutants do not support HDV infection, resulting in similar negative Delta Ag staining, which likely contributed to the oversight of this unfortunate error.

Upon a thorough re-examination of the raw microscopic data generated in this work and the presented images, we also noticed that: the image for Delta Ag*pcDNA6 in Figure 7A (the 1^st^ image on the left in the 2^nd^ row) partially overlaps with the Delta Ag staining image for “Huh-7/pcDNA6 (200×) ” in Figure 5B; and the image for Delta Ag*hNTCP in Figure 7A (the 3^rd^ image from the left in the 2^nd^ row) partially overlaps with the Delta Ag staining image for “Huh-7/hNTCP (200×) ” in Figure 5B. These two sets of images in Figure 7A and Figure 5B shared their respective source images as they were derived from the same batch experiment, and the presented images partially overlap. Unfortunately, we missed explicitly describing this in the legend during paper preparation.

We sincerely apologize for the oversight.

We have now provided the corrected Figure 7A, in which the image for Delta Ag*mkNTCP Q84R/N86K (the 4^th^ image from the left in the 4^th^ row) has been replaced with the image cropped from its own source image; the images for Delta Ag* pcDNA6 and Delta Ag* hNTCP are unchanged but along with an updated legend affirming the information of shared source image files in Figure 7A and Figure 5B, and a typo has been corrected from “7 dpi” to “8 dpi”.

The corrected Figure 7A legend is shown here:

**Identification of a critical region (aa 157–165) of NTCP for pre-S1 binding and viral infections**.

(**A**) Pre-S1 binding and HDV infection on cells expressing wild-type or mutant NTCPs. Corresponding amino acids (one-letter form) at the mutated positions of NTCP are shown for hNTCP, crab-eating monkey NTCP (mkNTCP), and tsNTCP. Huh-7 cells were transfected with plasmids encoding tsNTCP, hNTCP, mkNTCP, or NTCP mutants as indicated. The mutant NTCPs include hNTCP-bearing mutations of mkNTCP residues and mkNTCP-bearing mutations of human residues at indicated positions. The transfected cells were maintained in PMM for 24 hr and then either stained with 200 nM FITC-pre-S1**,** or infected with 500 mge HDV **under the same experimental conditions as described in Figure 5B**. HDV delta antigen in infected cells was detected with mAb 4G5 on **8** dpi. **The delta antigen staining images of pcDNA6 and hNTCP shared the same source images for Huh-7/pcDNA6 and Huh-7/hNTCP in Figure 5B, respectively**. Replacing aa 157–165 of mkNTCP with human counterpart rendered mkNTCP an efficient receptor for pre-S1 binding and HDV infection.

The original published Figure 7A legend is shown for reference:

**Identification of a critical region (aa 157–165) of NTCP for pre-S1 binding and viral infections**.

(**A**) Pre-S1 binding and HDV infection on cells expressing wild-type or mutant NTCPs. Corresponding amino acids (one-letter form) at the mutated positions of NTCP are shown for hNTCP, crab-eating monkey NTCP (mkNTCP), and tsNTCP. Huh-7 cells were transfected with plasmids encoding tsNTCP, hNTCP, mkNTCP, or NTCP mutants as indicated. The mutant NTCPs include hNTCP-bearing mutations of mkNTCP residues and mkNTCP-bearing mutations of human residues at indicated positions. The transfected cells were maintained in PMM for 24 hr and then either stained with 200 nM FITC-pre-S1 or infected with 500 mge HDV. HDV delta antigen in infected cells was detected with mAb 4G5 on 7 dpi. Replacing aa 157–165 of mkNTCP with human counterpart rendered mkNTCP an efficient receptor for pre-S1 binding and HDV infection.

The corrected Figure 7 is shown here:

**Figure 1:**
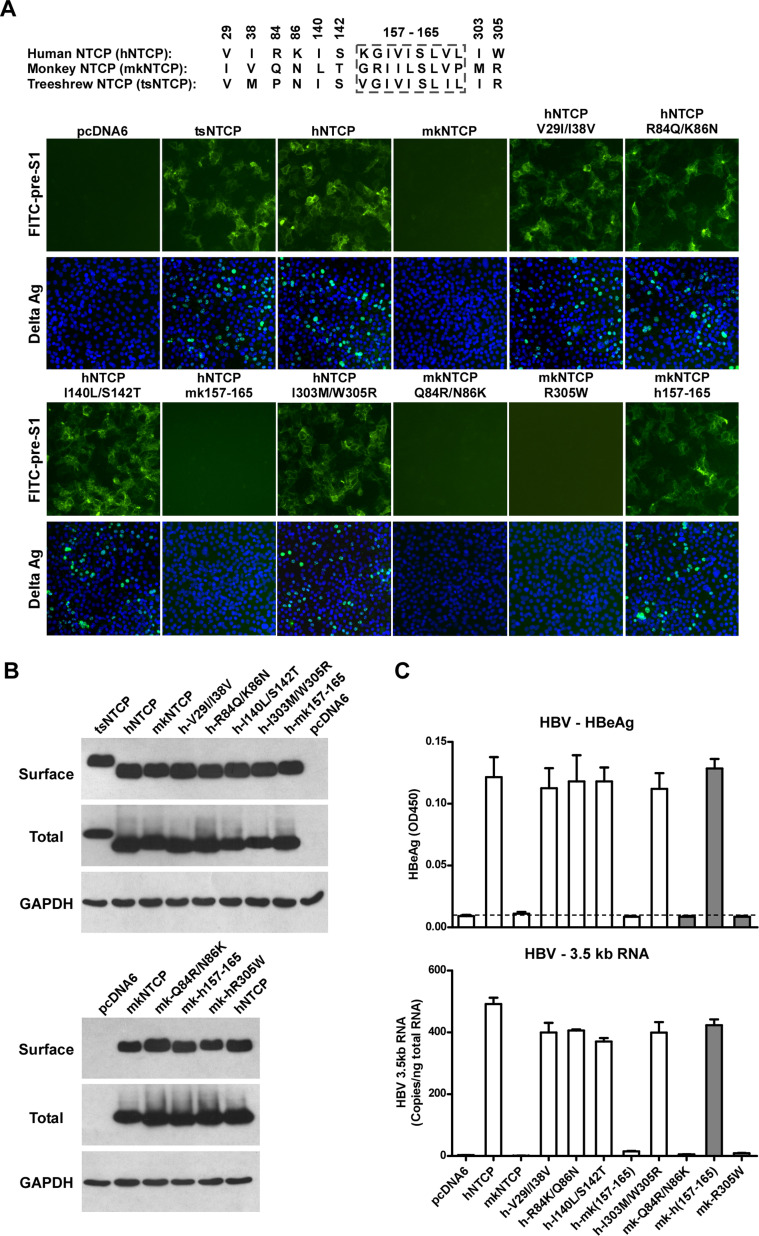


The original published Figure 7A legend is shown for reference:

**Figure 2:**
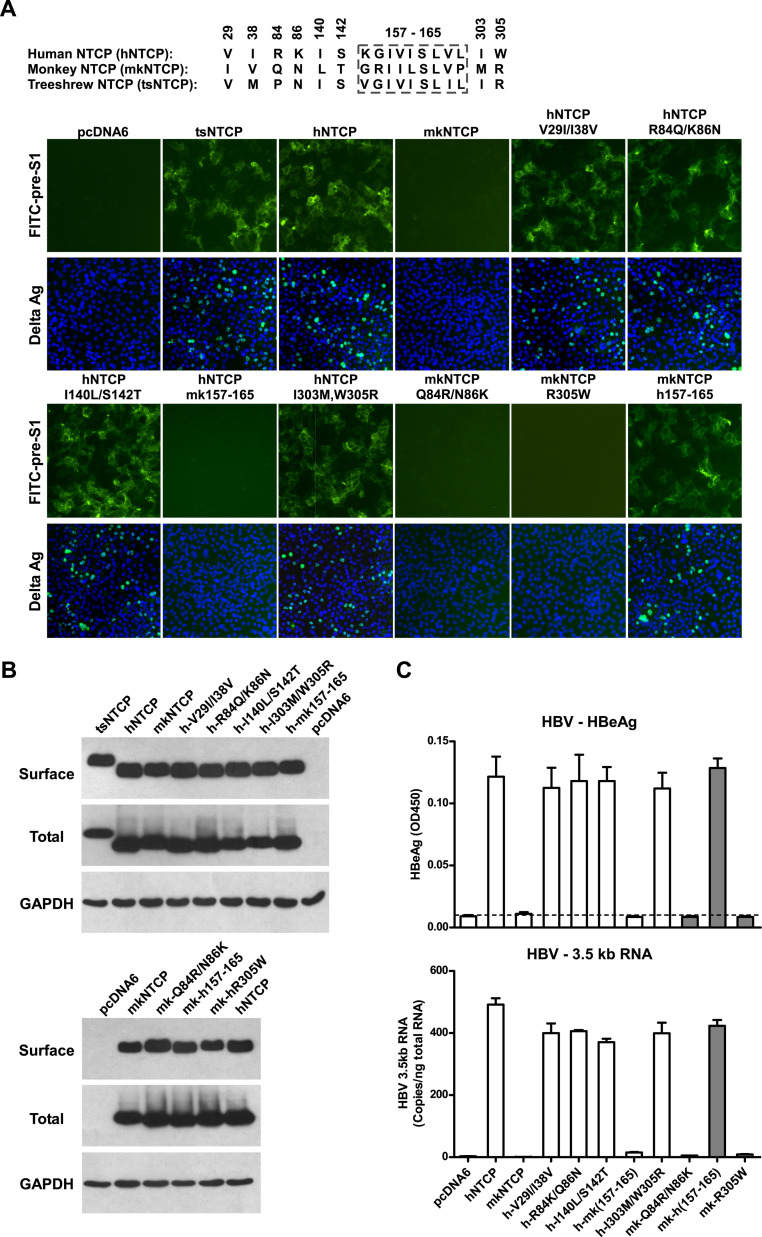


The article has been corrected accordingly.

