# Supplementary material for: Sodium taurocholate cotransporting polypeptide is a functional receptor for human hepatitis B and D virus
Source: eLife. 2012 Nov 13;1:e00049. doi: 10.7554/eLife.00049 (PMC3485615; doi:10.7554/eLife.00049)
Supplement: Figure 2—source data 1. — http://dx.doi.org/10.7554/eLife.00049.005 [file elife-00049-fig2-data1.pdf]

Figure 2 - Source Data 1

Top ten non-redundant proteins identified in the three samples by MS analysis

Sample	Name	Length (aa)	MolWt (Da)	Sequence Count	Spectrum Count	Functional Annotation	Sequence Coverage
65kDa – WT_b/PNGaseF (-) sample	GENSCAN_GS00000231568	209	22371	4	13	ref|NP_002761.1| trypsin-2 preproprotein [Homo sapiens]	14.80%
	comp5_c0_seq1|GENSCAN_predicted_ peptide_1|608_aa	608	68682	3	11	ref|NP_000468.1| 609 serum albumin preproprotein [Homo sapiens]	4.30%
	ENSTBEP00000000142	128	14403	2	13	ref|NP_003372.1| VIP peptides isoform 1 preproprotein [Homo sapiens]	9.40%
	comp7554_c0_seq2|GENSCAN_predicted_ peptide_1|379_aa	379	41483	2	8	ref|NP_003040.1| 349 sodium/bile acid cotransporter [Homo sapiens]	6.60%
	GENSCAN_GS00000235950	102	10754	1	1	ref|NP_004474.2| glycine cleavage system H protein, mitochondrial precursor [Homo sapiens]	21.60%
	comp376_c0_seq1|GENSCAN_predicted_ peptide_1|733_aa	733	84789	1	1	ref|NP_005339.3| 732 heat shock protein HSP 90-alpha isoform 2 [Homo sapiens]	2.50%
	comp59_c0_seq2|GENSCAN_predicted_ peptide_1|330_aa	330	37147	1	2	ref|NP_995584.1| 265 UDP-glucuronosyltransferase 1-6 isoform 2 [Homo sapiens]	2.40%
	GENSCAN_GS00000261263	802	84357	1	4	ref|NP_006112.3| keratin, type II cytoskeletal 1 [Homo sapiens]	1.50%
	GENSCAN_GS00000252787	713	73842	1	2	ref|NP_000412.3| keratin, type I cytoskeletal 10 [Homo sapiens]	1.30%
	comp218_c0_seq1|GENSCAN_predicted_ peptide_1|1178_aa	1178	129710	1	4	ref|NP_071504.2| 1178 pyruvate carboxylase, mitochondrial precursor [Homo sapiens]	1.20%
43 kDa – WT_b/PNGaseF (+) sample	GENSCAN_GS00000231568	209	22371	5	25	ref|NP_002761.1| trypsin-2 preproprotein [Homo sapiens]	12.90%
	comp7554_c0_seq2|GENSCAN_predicted_ peptide_1|379_aa	379	41483	3	6	ref|NP_003040.1| 349 sodium/bile acid cotransporter [Homo sapiens]	6.60%
	comp5_c0_seq1|GENSCAN_predicted_ peptide_1|608_aa	608	68682	8	32	ref|NP_000468.1| 609 serum albumin preproprotein [Homo sapiens]	6.20%
	comp218_c0_seq1|GENSCAN_predicted_ peptide_1|1178_aa	1178	129710	2	5	ref|NP_071504.2| 1178 pyruvate carboxylase, mitochondrial precursor [Homo sapiens]	2.00%
	ENSTBEP00000000142	128	14403	1	4	ref|NP_003372.1| VIP peptides isoform 1 preproprotein [Homo sapiens]	9.40%
	GENSCAN_GS00000279109	265	28676	1	2	~ref|NP_037362.1| signal transducer CD24 precursor [Homo sapiens]	7.90%
	ENSTBEP00000001232	676	75890	1	1	ref|NP_659001.2| sciellin isoform 1 [Homo sapiens]	6.70%
	GENSCAN_GS00000184315	322	34272	1	1	ref|NP_001989.2| fibulin-2 isoform b precursor [Homo sapiens]	5.30%
	GENSCAN_GS00000184883	238	25776	1	1	Uncharacterized protein	2.90%
	comp5222_c0_seq1|GENSCAN_predicted_ peptide_1|1285_aa	1285	148568	1	1	ref|NP_004990.3| 1285 myosin-VI [Homo sapiens]	1.50%
43 kDa – $N9K_b$/PNGaseF (+) sample	GENSCAN_GS00000203437	578	61569	14	45	ref|NP_005546.2| keratin, type II cytoskeletal 6B [Homo sapiens]	15.60%
	GENSCAN_GS00000261263	802	84357	10	46	ref|NP_006112.3| keratin, type II cytoskeletal 1 [Homo sapiens]	6.50%
	GENSCAN_GS00000261502	378	40781	8	24	ref|NP_000517.2| keratin, type I cytoskeletal 14 [Homo sapiens]	20.10%
	GENSCAN_GS00000252787	713	73842	8	24	ref|NP_000412.3| keratin, type I cytoskeletal 10 [Homo sapiens]	7.90%
	ENSTBEP00000007380	537	59027	6	28	ref|NP_000414.2| keratin, type II cytoskeletal 2 epidermal [Homo sapiens]	7.10%
	comp5_c0_seq1|GENSCAN_predicted_ peptide_1|608_aa	608	68682	6	24	ref|NP_000468.1| 609 serum albumin preproprotein [Homo sapiens]	3.90%
	GENSCAN_GS00000214315	505	55293	5	13	ref|NP_005548.2| keratin, type I cytoskeletal 16 [Homo sapiens]	9.10%
	GENSCAN_GS00000261260	1169	125254	4	16	ref|NP_778253.2| keratin, type II cytoskeletal 1b [Homo sapiens]	3.20%
	GENSCAN_GS00000211249	815	90386	4	13	ref|NP_002266.2| keratin, type I cytoskeletal 15 [Homo sapiens]	3.70%
	GENSCAN_GS00000223539	804	89223	4	7	ref|NP_000415.2| keratin, type II cytoskeletal 5 [Homo sapiens]	4.60%

Name: target protein name in the database. **Length**: number of amino acids. **MolWt**: calculated molecular weight (Dalton). **Sequence Count**: distinct peptides identified in a protein. **Spectrum Count**: number of spectrum identified in MS/MS. **Functional Annotation**: annotation of the protein according to their homolog in human. **Sequence Coverage**: percentage of the protein sequences that covered by identified peptides.
